# On Conflicts between Pharmaceutical Patent Protection and the Right to Life and Health Based on a Stackelberg Game

**DOI:** 10.3390/ijerph18031119

**Published:** 2021-01-27

**Authors:** Chunming Xu, Debao Zhu

**Affiliations:** 1 Shanghai International College of Intellectual Property, Tongji University, Shanghai 200092, China; romin888@sina.com; 2 School of Management, Shanghai University, Shanghai 200444, China

**Keywords:** pharmaceutical patent, public health, excess profit tax, Stackelberg game

## Abstract

To solve the conflict between pharmaceutical patent protection and the right to life, health and increased patient satisfaction, drug prices can be regulated by levying an excess profit tax. An optimal tax strategy was formulated that not only could lower drug prices and improve public health and welfare, but also considers companies’ earnings. The strategy was based on the Stackelberg game theory as a bi-level mathematical model. In the model, the government is the leader, with patient satisfaction as the main goal, and pharmaceutical companies are the followers, with maximum drug revenue as the goal. The results show that under the premise of ensuring sufficient incentives for patent holders, the optimized tax on excess profit can effectively compensate for the shortcomings of pharmaceutical patent protection, alleviate the failure of market regulation of drug prices, improve patient satisfaction, and increase total social welfare.

## 1. Introduction

Under the current system, there is an irreconcilable conflict between pharmaceutical patent protection and drug accessibility. On the one hand, the research and development (R&D) process for drugs is long and complex, with high investment and high risk; therefore, without pharmaceutical patent protection, pharmaceutical companies do not have the motivation to carry out R&D, which eventually leads to medical technology stagnation and brings great harm to public health [1,2]. On the other hand, pharmaceutical patent protection inevitably gives patent holders a certain monopoly power, and drug prices are often set by the patent holders; therefore, there is the possibility of abuse of the dominant position to manipulate the market. Driven by seeking profit, pharmaceutical companies with monopoly rights set drug prices significantly higher than the overall costs. For example, the prices of Gleevec and Tasigna (Novartis), Tarceva (Roche), Sutent (Pfizer), Nexavar (Bayer), and Revlimid (Celgene) are 4–12 times the price of their generic drug counterparts [3]. In the United States (US), which has strong patent protection, the prices for some drugs are much higher than those for the same drugs in the United Kingdom (UK), where they are priced based on cost-effectiveness [4,5] (as shown in Table 1).

All these issues have resulted in an irreconcilable conflict between pharmaceutical patent protection and drug accessibility, contradicting the original intention of pharmaceutical patent protection.

## 2. Review of the Relevant Literature

### 2.1. Existing Countermeasures to Resolve Conflicts

The pharmaceutical industry has high patent layout capabilities and strong patent lifecycle management abilities and often files offensive patents at later stages of the life cycle to extend the patent protection for new drugs; therefore, there is an ever-increasing number of patents applications for each drug, creating a patent fence [6]. The patent applied by the Wuhan Institute of Virology, Chinese Academy of Sciences, is a typical surrounding patent for pharmaceutical use. For pharmaceutical companies with R&D strength, applying for surrounding patents for pharmaceutical use mainly serves to extend the patent term for patented drugs; for generic pharmaceutical companies, applying for surrounding patents for pharmaceutical use allows them to bypass a pharmaceutical patent and increase their market value and share. Peng et al. [7] analyzed the formulation purpose and original protection intention of patents for pharmaceutical use, extracted the technical characteristics that may affect the protection scope of this type of patent, determined the influencing factors, and proposed ideas for defining the protection scope of patents for pharmaceutical use. Sternitzke et al. [6] studied the mechanisms of patent protection on new drugs, including the application methods, the time needed for granting a patent, and the skills required to achieve profit maximization based on patent legislation and the use of the patent system. Together with the inherent backwardness of the patent system, the system is not able to maximize total social welfare. 

### 2.2. Existing Countermeasures for Resolving Conflicts

To resolve conflicts between drug accessibility and pharmaceutical patent protection, there are two approaches that are commonly used: (A) compulsory licensing, parallel imports at the pricing stage, and other means [2,8,9]; these methods restrict patent rights and reduce drug prices from the perspective of breaking monopolies; and (B) the implementation of medical insurance policies [10,11]; this measure targets the sales process, directly reduces the actual payments by patients, and transfers the patients’ partial burden of drugs to other organizations, such as the government, thus improving patient satisfaction. 

#### 2.2.1. Compulsory Licensing

Compulsory licensing has been used in some developing countries and has been effective in suppressing drug prices, but whether it stifles innovation is debatable. For example, India’s compulsory licensing system provides people with reliable and stable drug prices, and its 2005 Patent Law also stimulates innovation [12,13]. China also has a compulsory licensing system for patents [14]; however, China is still very cautious about the implementation of compulsory licensing. Chen et al. [15] studied drugs, such as Tamiflu, used for the prevention and control of the Influenza A/H1N1 epidemic and noted several following reasons for not enforcing compulsory licensing in China. Compulsory licensing has some technical obstacles; the application of China’s intellectual property (IP) system needs to be improved, and there is tremendous pressure from foreign companies and even foreign governments [16,17]. In fact, most developing countries impose compulsory licensing in specific fields. Bond et al. [18] analyzed how the threat of price control and compulsory licensing affects consumers in developing countries (south) obtaining foreign patented products. Regarding the claim that compulsory licensing for drugs from developed countries would weaken technological innovation, developing countries argue that little or even no compensation would not impede technological development. However, compulsory licensing controls the sales prices of drugs from developed countries in developing countries, inevitably weakening the incentive for pharmaceutical companies to invest in R&D. Therefore, compulsory licensing should be strictly restricted and is not the optimal choice to resolve the conflict between pharmaceutical patent protection and the right to life and health.

#### 2.2.2. Medical Insurance

To solve the problem of high drug prices, in addition to pricing controls, various countries have also adopted different medical insurance systems, such as commercial medical insurance systems, national health insurance systems, and social medical insurance systems, to protect patients’ right to health. Insurance companies in the US actively negotiate with pharmaceutical companies to keep drug prices as low as possible. After obtaining approval for drug prices, Canadian pharmaceutical companies must further apply to the Canadian Drug Expert Committee (CDEC) to include the drugs in the reimbursement catalog, and then, drug prices are determined after consultation. The basic medical insurance system in China is a risk-sharing system; that is, according to the established reimbursement percentages, where the insurance companies directly help patients pay part of drug prices to reduce the financial burden of patients [19,20]. In recent years, to further reduce the financial burden of patients for medical care, some provinces, such as Zhejiang and Jiangsu, have lowered drug prices through tripartite negotiations among health insurance companies, medical institutions, and pharmaceutical suppliers and have included patented drugs for the treatment of some serious diseases, major diseases and chronic diseases in the medical insurance payment system through some medical insurance policies, such as catastrophic medical insurance.

Although the abovementioned methods can control drug prices and meet the urgent needs of patients, the scope of price control is limited, the types of drugs that can be controlled are limited, and government finances are limited. Methods of solving conflicts from various aspects more scientifically and rationally remain to be explored.

## 3. Research Hypotheses and Model Design

### 3.1. Basic Description and Assumptions of the Model

Game theory is a decision-making theory used to deal with problems characterized by conflict. Starting from the essence of conflict, game theory makes a general abstract description of each subject that has a competitive relationship. The leader–follower game model was first put forward when studying the disequilibrium economic market competition. Since the 1980s, it has been inspired by the game theory of the German economist Stackelberg [4], which has attracted widespread attention from scholars. The leader–follower game model studies the orderly and non-cooperative interaction between two decision makers with each objective function. The upper-level leader gives priority to making decisions, and the follower at the lower level responds to its interests under the decision-making information from the upper level. One participant’s behavior affects the other’s strategic choices and the realization of the goal, and neither side can completely control the other’s choice behavior, therefore, the upper-level decision-maker should ultimately make a final decision that conforms to its interests based on the lower-level responses. This reciprocation finally reaches a decision-making plan that both upper-level leaders and lower-level followers are satisfied with. Consider the Stackelberg–Nash-Cournot leader–follower game model as follows;

$$\begin{array}{l} \underset{x,y}{\min}\;F\left(x,y\right)\\ s.t.\;G\left(x\right)\leq 0,\\ \begin{array}{cc} & \underset{y}{\min}\;f\left(y\right) \end{array}\\ \begin{array}{cc} & s.t.\;g\left(y\right) \end{array}\leq 0 \end{array}$$

The structure of the leader–follower game model is very complicated. Even the simplest linear problem has been proved to be NP-hard, and generally speaking, it is also a non-convex optimization problem. The process of getting the optimal solution is very difficult. Even if the solution can be found, it is usually only a local optimal solution rather than a global one. It is often difficult to obtain a satisfactory optimal solution through general methods. Karush–Kuhn–Tucker Conditions (KKT condition) is typically used in transforming the leader–follower game into a single-level problem to solve in the existing researches.

The conflict between drug accessibility and pharmaceutical patent protection cannot be solved in the profit-oriented free market; therefore, the government is obligated to formulate policies to guide the continued development of the pharmaceutical industry. The policies can not only protect the public’s rights to use drugs and improve drug accessibility but can also provide sufficient incentive to pursue continuous innovation. This paper proposes the use of an excess profit tax to influence the tax burden of pharmaceutical companies to control excessively high drug prices. Additionally, companies’ decisions regarding the excess profit tax could also reversely affect the government, mutually restraining the two. This is a typical multi-interest optimization problem, therefore, the decision-making position and the behavior between the government and pharmaceutical companies are consistent with the bilevel game model, so this model can be used to describe the interactions between government agencies and pharmaceutical companies. The government is the leader prior to the decision-making of pharmaceutical companies, and the behaviors of the two entities influence and restrict each other. This model is used to achieve the following two aims:

1. To determine the premium ratio for drug prices when the government should start to levy an excess profit tax on pharmaceutical companies and the optimal tax rate and, based on market conditions, to determine the reimbursement percentage for drugs through medical insurance, which can effectively improve patient satisfaction; and
2. To determine how to price various drugs and how to make corresponding production plans under this tax system to maximize the interests of pharmaceutical companies.

The following are the basic assumptions of the model:

- The threshold of the excess profit tax is known, and the tax is a fixed-rate tax;
- The study period is based on the patent term of the core drug compounds, and the market condition after the patent expires is not considered;
- The number of pharmaceutical companies with patented drugs is reduced to one;
- The types of drugs are assumed to be n, and the quality of each drug (n drugs) is directly proportional to the price;
- The drug price is inversely proportional to the drug yield and patient satisfaction; and
- The tax rate is proportional to the amount of excess profit.

The parameters involved in this model are shown in Table 2.

### 3.2. The Stackelberg Game Model of the Government and Companies

#### 3.2.1. Lower-Level Pharmaceutical Companies

The parameters used in modeling the lower-level pharmaceutical companies are as follows. In the study period, the drug yield of the ith drug (i=1,…,n) is $y_{i}$ ($y_{i}=\left(y_{1},\ldots ,y_{n}\right)$, $y_{i}\geq 0$), the unit cost of the drug, including R&D costs, is $c_{i}$, the drug price is $p_{i}$, the tax rate for excess profit by the government is *t* (0 ≤ *t* ≤ 1), the premium ratio for a drug price is $\alpha_{i}$ ($\alpha_{i}>1$), and the income for pharmaceutical companies from drug sales is *E*_1_

$$E_{1}=\sum_{i=1}^{n}\left(\alpha_{i}-1\right)\cdot c_{i}\cdot y_{i}.$$

The excess profit that needs to be taxed is *E*_2_ (0 ≤ *E*_2_ ≤ *E*_1_):

$$E_{2}=E_{1}-E_{0}.$$

Since the production cost is fixed, let the total cost be C; then

$$C=\sum_{i=1}^{n}c_{i}\cdot y_{i},$$

There is a certain inverse relationship between the drug price $p_{i}$ and market demand. This paper assumes that drugs produced by pharmaceutical companies are not necessary and irreplaceable drugs and that the reimbursement percentage for the ith drug is $\sigma_{i}$; therefore, the actual selling price of the drug (namely, the actual price paid by the patient) is

$$p_{i}=\alpha_{i}\cdot c_{i}\cdot \left(1-\sigma_{i}\right).$$

The drug yield $y_{i}$ is related to the market supply and demand, and the model uses an inverse proportional function

pi=f(yi)=aiyi (ai>0).

The optimization model for lower-level decision makers (pharmaceutical companies) to optimize their own interests is

(1) $$\underset{\alpha_{i},y_{i}}{\max}\sum_{i=1}^{n}E_{1}^{i}-E_{2}^{i}\cdot t$$

(2) $$\begin{array}{cc} s.t. & p_{i}=f(y_{i}), \end{array}$$

(3) $$y_{i}=\log_{d}\alpha_{i},$$

(4) $$E_{1}^{i}=\left(\alpha_{i}-1\right)\cdot c_{i}\cdot y_{i}=\left(p_{i}-c_{i}\right)\cdot y_{i},$$

(5) $$E_{2}^{i}=\left\{ \begin{array}{l} 0,others\\ E_{1}^{i}-E_{0,}\;if\;E_{1}>E_{0},\;and\;\alpha >2 \end{array}\right.,$$

(6) $$\alpha_{i}\geq 1,\;c_{i}\geq 0,\;0\leq t\leq 1,\;i=1,\ldots ,n.$$

Among these, Equation (1) is the objective function of the pharmaceutical company, which represents the maximum profit. Constraints (2) and (3) are the market restrictions on the production and sales price of patented drugs within the study period. Constraint (4) describes the revenue function of the pharmaceutical company. Constraint (5) describes the government’s tax amount function. Constraints (6) describe the premium ratio of various drugs, costs of various drugs, and government tax rate, respectively. The overpricing by pharmaceutical companies can not only result in losses in market share to generic pharmaceutical companies but also increase their own tax burden.

#### 3.2.2. Upper-Level Government Agencies

The parameters used in modeling the upper-level government agencies are as follows:

Patient satisfaction is described with s and is calculated using a 100-point scale:

$$s_{i}=\left(s_{1},\ldots ,s_{n}\right).$$

Patient satisfaction $s_{i}$ and tax revenue are weighted and averaged by $\beta_{j}$ (j=1,2,3), and the sum is used as the optimization target by upper-level government agencies. Patient satisfaction $s_{i}$ and drug price $p_{i}$ satisfy an inverse relationship, i.e., patient satisfaction with ith drug is as follows:

si=g(pi)=bipi (bi>0).

In addition, as mentioned above, government tax revenue is $E_{2}t$, and government health insurance expenditure is

$$\sum_{i=1}^{n}E_{1}^{i}\cdot \sigma_{i}.$$

Additionally, *E*_2_ and *t* are related to each other. According to the principle of taxation, the higher the income is, the higher the tax rate. The tax rate *t* and the excess profit *E*_2_ satisfy the following relationship

$$t=h\left(E_{2}^{i}\right).$$

The government must first consider public health interests, i.e., the general population’s satisfaction with drug prices. Therefore, the government’s decision-making model is

(7) $$\underset{\alpha_{i},t,y_{i}}{\max}\beta_{1}\sum_{i=1}^{n}s_{i}+\beta_{2}t\sum_{i=1}^{n}E_{2}^{i}-\beta_{3}\sum_{i=1}^{n}E_{1}^{i}\cdot \sigma_{i}$$

(8) $$\begin{array}{cc} s.t. & s_{i}=g\left(p_{i}\right), \end{array}$$

(9) $$t=h\left(E_{2}^{i}\right),$$

(10) $$\beta_{1}+\beta_{2}+\beta_{3}=1,$$

(11) $$0\leq \beta_{j}\leq 1,j=1,2,3\;,$$

(12) $$p_{i}=\left(1-\sigma_{i}\right)\alpha_{i}\cdot c_{i}$$

Among them, Equation (7) is the government’s objective function, which consists of patient satisfaction, tax revenue, and medical insurance expenditure, where the contribution of medical insurance expenditure to the government’s goal is negative. Constraint condition (8) represents the influence of drug price on patient satisfaction, and constraint condition (9) represents the influence of excess profit on the taxation rate. The higher the price of drugs, the higher the excess profits, followed by the higher tax rates are needed to weaken the motivation of pharmaceutical companies and to increase pricing. Constraint (10) describes the distribution ratio of the upper government in the three parts of satisfaction, tax revenue, and medical insurance expenditure. Constraint (11) describes the range of the coefficients of the government’s three goals mentioned in (10). Constraint (12) represents the price of medicine actually paid by the patient.

Therefore, the bi-level planning model of government agencies and pharmaceutical companies under an excess profit tax policy is

$$Upper\;level\;decision-making\;model\left\{ \begin{array}{l} \underset{\alpha_{i},t,y_{i}}{\max}\beta_{1}\sum_{i=1}^{n}s_{i}+\beta_{2}t\sum_{i=1}^{n}E_{2}^{i}-\beta_{3}\sum_{i=1}^{n}E_{1}^{i}\cdot \sigma_{i}\\ s.t.\\ s_{i}=g\left(p_{i}\right),\\ t=h\left(E_{2}^{i}\right),\\ \beta_{1}+\beta_{2}+\beta_{3}=1,\\ 0\leq \beta_{j}\leq 1,j=1,2,3\;. \end{array}\right.$$

$$Lower\;level\;decision-making\;model\left\{ \begin{array}{l} \underset{\alpha_{i},y_{i}}{\max}\sum_{i=1}^{n}E_{1}^{i}-E_{2}^{i}\cdot t\\ s.t.\\ p_{i}=f(y_{i}),\\ y_{i}=\log_{d}\alpha_{i},\\ E_{1}^{i}=\left(\alpha_{i}-1\right)\cdot c_{i}\cdot y_{i}=\left(p_{i}-c_{i}\right)\cdot y_{i},\\ E_{2}^{i}=\left\{ \begin{array}{l} 0,\;others\\ E_{1}^{i}-E_{0,}\;if\;E_{1}>E_{0},\;and\;\alpha >2 \end{array}\right.,\\ \alpha_{i}\geq 1,c_{i}\geq 0,0\leq t\leq 1,i=1,\ldots ,n. \end{array}\right.$$

It can be seen from models (I) and (II) that both drug price and the amount of excess profit impose constraints on government decisions, and that drug price is directly related to patient satisfaction. Both players in the game must consider each other’s expected bottom line when achieving their maximum benefits.

The determination of excess profit $E_{2}$ by the government requires extensive social investigation in order to determine the tax threshold. If $E_{2}$ is too large, the company’s enthusiasm for R&D could be affected, innovation processes for new drugs and related technologies could be hindered, and the interests of all of society could be harmed. If $E_{2}$ is too small, on the one hand, the goal of curbing high-priced drugs and improving patient welfare cannot be attained; on the other hand, the increase in fiscal revenue will be limited. It is also critical to determine the tax rate *t* for excess profit; the process for determining *t* is similar to that for $E_{2}$.

### 3.3. Model Transformation

The Lagrange multiplier method and Karush–Kuhn–Tucker (KKT) condition are two very important methods for solving constrained optimization problems. For optimization problems with equality constraints, the Lagrange multiplier method can be used to obtain optimal values. For optimization problems with inequality constraints, the KKT condition can be used to obtain optimal values. For obtaining results, these two methods utilize only the necessary conditions, and only in the case of convex functions can the sufficient and necessary conditions be guaranteed. The KKT condition is the generalized Lagrange multiplier method [21,22].

Equivalent transformation of the lower-level optimization model was performed using the KKT condition, and the augmented Lagrange function *L* of the lower-level objective function $\sum_{i=1}^{n}E_{1}^{i}-E_{2}^{i}\cdot t$ is

$$\begin{array}{l} L=\sum_{i=1}^{n}\left(E_{1}^{i}-E_{2}^{i}\cdot t\right)+\sum_{i=1}^{n}\left(u_{i}^{1}\right)\left(1-\alpha_{i}\right)+\sum_{i=1}^{n}\left(u_{i}^{2}c_{i}\right)+\nu_{1}\left(-t\right)+\nu_{2}\left(t-1\right)\\ =-\sum_{i=1}^{n}\left[\left(\alpha_{i}-1\right)\cdot c_{i}\cdot y_{i}\cdot \left(1-t\right)\right]+\sum_{i=1}^{n}\left[u_{i}^{1}\left(1-\alpha_{i}\right)+\left(u_{i}^{2}c_{i}\right)\right]+E_{0}t. \end{array}$$

Thus,

$$\begin{array}{l} \underset{\alpha_{i}}{\nabla }L=-c_{i}\cdot y_{i}\cdot \left(1-t\right)-u_{i}^{1},\\ \underset{y_{i}}{\nabla }L=-\left(\alpha_{i}-1\right)\cdot c_{i}\cdot \left(1-t\right). \end{array}$$

Therefore, the KKT condition for the lower-level model is:

$$\begin{array}{l} \underset{\alpha_{i}}{\nabla }L=-c_{i}\cdot y_{i}\cdot \left(1-t\right)-u_{i}^{1}=0,\;i=1,\ldots ,n,\\ \underset{y_{i}}{\nabla }L=-\left(\alpha_{i}-1\right)\cdot c_{i}\cdot \left(1-t\right)=0,\;i=1,\ldots ,n,\\ u_{i}^{1}\left(1-\alpha_{i}\right)=0,\;i=1,\ldots ,n,\\ u_{i}^{2}c_{i}=0,\;i=1,\ldots ,n,\\ \nu_{1}\left(-t\right)=\nu_{2}\left(t-1\right)=0,\\ u_{i}^{1},u_{i}^{2},\nu_{1},\nu_{2}\geq 0 \end{array}$$

Therefore, the Stackelberg game model can be transformed into the following form,

$$\begin{array}{l} \underset{\alpha_{i},t,y_{i},u_{i}^{1},u_{i}^{2},\nu_{1},\nu_{2}}{\max}\beta_{1}\sum_{i=1}^{n}s_{i}+\beta_{2}\sum_{i=1}^{n}\left[\left(\alpha -1\right)c_{i}y_{i}-E_{0}\right]t-\beta_{3}\sum_{i=1}^{n}\left(\alpha -1\right)c_{i}y_{i}\sigma_{i}\\ s.t.\\ s_{i}=g\left(p_{i}^{\prime }\right),\\ t=h\left(E_{2}^{i}\right),\\ \beta_{1}+\beta_{2}+\beta_{3}=1,\\ 0\leq \beta_{i}\leq 1,\;i=1,2,3\;,\\ p_{i}^{\prime }=\left(1-\sigma_{i}\right)\alpha_{i}\cdot c_{i},\\ -c_{i}\cdot y_{i}\cdot \left(1-t\right)-u_{i}^{1}=0,\\ \underset{y_{i}}{\nabla }L=-\left(\alpha_{i}-1\right)\cdot c_{i}\cdot \left(1-t\right),\;i=1,\ldots ,n,\\ u_{i}^{1}\left(1-\alpha_{i}\right)=0,\;i=1,\ldots ,n,\\ u_{i}^{2}c_{i}=0,\;i=1,\ldots ,n,\\ \nu_{1}\left(-t\right)=\nu_{2}\left(t-1\right)=0,\\ u_{i}^{1},u_{i}^{2},\nu_{1},\nu_{2}\geq 0,\;i=1,\ldots ,n. \end{array}$$

Next, the solutions of the transformed model (III) were obtained.

## 4. Results and Discussion

### 4.1. Results

When setting up numerical examples in MATLAB 2016a [23], in the calculation cycle, the unit cost of drugs includes all costs, such as R&D investment, machinery and equipment, and labor (dividing all costs by the number of drugs (box)). The total investment by the company was 5 million RMB, and only two drugs were produced, denoted as D1 and D2. It was assumed that the cost of D1 was 15 RMB, and that of D2 was 20 RMB. Patient satisfaction was evaluated using a 100-point scale (0–100), and the excess profit tax could be levied when the sales volume of the patented drug reaches 100,000 RMB.

**Figure 1:**
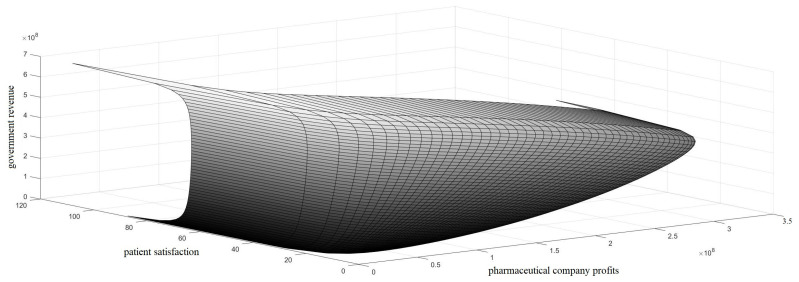
The interests of the government, pharmaceutical companies, and patients.

As shown in Figure 1, the x-axis represents the profits of the pharmaceutical company, the y-axis represents patient satisfaction, and the z-axis represents government tax revenue. The degree of satisfaction of the three stakeholders on their expected goals was classified as high, medium, and low. The practical dilemma corresponds to the region where (x, y, z) = (high, low, medium) in Figure 1. At this point, the profit for the pharmaceutical company was high, and patient satisfaction was low; the aim of this study was to break this status quo. In Figure 1, there is also a solution of (x, y, z) = (low, high, high/medium). In this case, the earnings of the company were not in line with the efforts, and the sustainable development of innovation is difficult to maintain. Therefore, the ideal solution should satisfy the following conditions: the company is guaranteed to receive certain profit incentives, drug prices (i.e., the premium ratio of drug price) should be reduced, and the drug supply (i.e., drug yield) should be guaranteed, i.e., (x, y, z) = (medium, high, medium/low); thus, drug accessibility is effectively improved.

In theory, including patented drugs into a medical insurance policy and levying an excess profit tax on drugs can effectively lower drug prices and reduce the financial burden on patients. However, to give the full play to this policy, the following details should be considered: (1) the types of drugs included in medical insurance policies and the percentage of reimbursement through medical insurance should be determined based on the government’s budget; and (2) the tax rate for the excess profit tax should be determined considering a balance between the development of the market economy and public interests. In particular, the relationship among the tax rate, drug yield, and the premium ratio for drug prices should be discussed in detail to determine the optimal solution for the policy so that its desired role is achieved.

**Figure 2:**
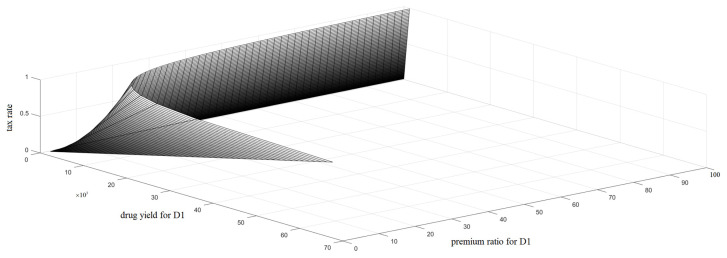
Relationships among tax rate and drug yield for D1 and the premium ratio for D1.

**Figure 3:**
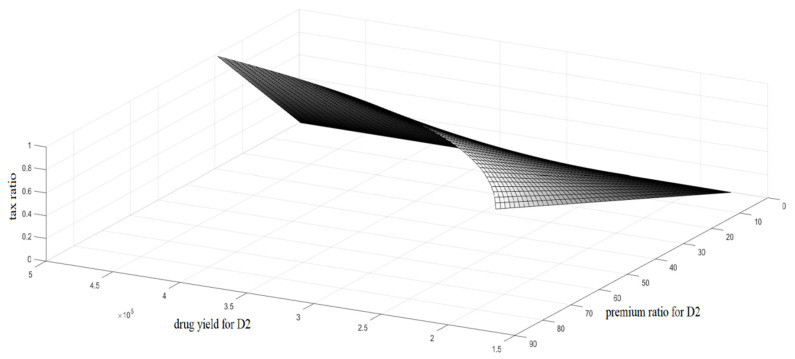
Relationships among tax rate and drug yield of D2, and the premium ratio of D2.

Next, the quantitative relationship among the tax rate for excess profit tax, drug yield, and the premium ratio for drug prices is analyzed, as shown in Figure 2 and Figure 3.

Figure 2 and Figure 3 further analyze the relationship among the tax rate for the excess profit tax, drug yield, and the premium ratio for drug prices; drug yield and premium ratio are two important factors that affect drug accessibility.

First, a high tax rate is not optimal. Theoretically, a high tax rate for excess profit can effectively curb the price of patented drugs; however, in the short term, a high tax rate could cause a shortage of drug supply (i.e., drug yield). As shown in Figure 2, when the tax rate is greater than 0.5, the output of drug 1 is always less than 10 × 10^3^; in Figure 3, the output of drug 2 also shows a downward trend under the high tax rate. In the long term, it could discourage companies from investing in R&D and affect the sustainable development of pharmaceutical technologies. Second, the pharmaceutical company in this model mainly makes price decisions regarding two drugs, and the cost of D2 is higher than that of D1.

Further, we analyzed the solutions in the higher patient satisfaction interval, as shown in Table 3. When patient satisfaction was more than scores 60, the government’s financial revenue was not proportional to the profits of the lower-level company, which indicates that there was a certain tax rate. Furthermore, the pricing strategy could take into account patient satisfaction, corporate profits, and government revenue, which does not require the higher price or, the lower tax rate. When a certain equilibrium was reached, the patients, the government, and the enterprise all reached the optimal state.

According to the results of Figure 2 and Figure 3, the pharmaceutical company is more inclined to produce high-cost drugs, which seems to be contrary to common sense. In fact, one of the implied assumptions of this model is that D1 and D2 are not equivalent in treatment efficacy. For high-cost D2, the premium ratio in the market is also high; that is, its added value is high, and the absolute profit per unit product is also high. In addition, considering that the government’s tax policies have a great impact on the market economy, especially on the decision-making of companies, it is necessary to be prudent regarding tax rates. Therefore, for effective tax rates for excess profit, the minimum tax rate should be taken to maximally weaken the impact of regulatory policies on the market.

At this point, after comprehensively considering the interests of the patients, the government, and the pharmaceutical company and optimizing the government’s tax policy on excess profit and medical insurance reimbursement policies, the optimal solution under equilibrium is obtained: the tax rate for excess profit t=42%, percentage of reimbursement through medical insurance $\sigma_{1}=0.40$ and $\sigma_{2}=0.40$, the premium ratio $\alpha_{1}=72$ and $\alpha_{2}=85$, and the drug yield $y_{1}=100,000$ and $y_{2}=175,000$.

At this point, the quantified result for patient satisfaction is s=98. Thus, the excess profit tax policy and the medical insurance reimbursement policy make substantial contributions to effectively controlling the prices of patented drugs and increasing drug accessibility. Furthermore, the company’s earnings are maintained at a relatively high level, and the high earnings are conducive to stimulating continuous pharmaceutical R&D investment in this field. However, under this optimal solution, the government has a high fiscal expenditure.

### 4.2. Discussion and Analysis

There is also another optimal solution in this model, that is, a mathematical solution with high patient satisfaction, and government revenue and low company earnings. From the patient’s point of view, this is undoubtedly the optimal solution. Even from the perspective of the government, this result is also quite ideal from a static view. However, it should be noted that this situation has obvious shortcomings and is contrary to the basic law of “labor-benefit”. In this situation, companies’ enthusiasm for continuous R&D is severely inhibited, which undoubtedly impedes the long-term development of society and neglects the interests of future patients. Therefore, such a situation is not a real optimal solution and has extremely unstable defects.

Therefore, in the process of finding an optimal solution, centering on improving drug accessibility and patient satisfaction, various factors should be comprehensively considered. Although this model has no parameter that directly characterizes drug quality, there are often quality differences between patented drugs and similar generic drugs. Because of patent effects, the general idea in the market is that the quality of patented drugs is superior to that of similar generic drugs. Based on this premise and combined with the “price-quality” effect, it can be seen that the higher a drug price is, the better the drug quality in the patient’s opinion. However, for drugs, which are a special commodity, the price sensitivity of patients is not high, and even for specific diseases or drugs within a certain price range, a drug price increase could weaken the incentive for patients to choose similar generic drugs. This is also consistent with this model’s solution. For D1 and D2, under the premise of the same percentage of reimbursement through medical insurance, the drug yield for D2 with a higher premium ratio (yield is positively correlated with market demand) is 1.75 times that for D1. Therefore, for drugs, an appropriately high price can promote sales, which is one of the hidden reasons that drug prices are often much higher than marginal costs. The nature of pharmaceutical companies’ pursuit of maximizing their own interests presumes that they have no motivation to improve patient satisfaction, drug accessibility, and public welfare; therefore, it requires the government to reasonably supervise drug prices through administrative means and guide the prosperous development of the overall pharmaceutical market.

Taxation on pharmaceutical companies’ income directly through an excess profit tax is highly efficient and simple. However, it is necessary to overcome the prejudice of one-sided justice and prudently set the tax rate so as to ensure that it can correct the social and public losses caused by monopolies, enable the patent holders to obtain sufficient incentives, and solve the existing problems while considering the future development of the medical field. In this tax policy setting, for pharmaceutical companies, increasing the premium ratio for drug prices can increase the profit per drug unit, but the tax burden and the loss of the patient market could reduce total revenue. Therefore, compared with compulsory licensing and other approaches, using a tax tool as a supplement to the existing patent system can effectively coordinate the conflict between drug accessibility and pharmaceutical patent protection. Additionally, reimbursement for drugs within a reasonable range can increase patients’ demand elasticity to curb drug price gouging by pharmaceutical companies and guide reasonable pricing by pharmaceutical companies. Second, financial subsidies can be used to support relevant pharmaceutical companies to ensure their profit margins, thus effectively encouraging pharmaceutical companies to invest in R&D and promote the sustainable development of the industry.

## 5. Conclusions

To solve conflicts between pharmaceutical patent protection and drug accessibility and increase patient satisfaction, this study establishes an excess profit tax policy on the basis of existing drug price control methods, such as compulsory licensing, parallel imports and medical insurance reimbursement policies, to regulate drug prices and formulates an optimal tax strategy to maximally reduce drug prices and improve the level of public health benefits, while considering companies’ earnings. First, this study establishes a bi-level mathematical model based on the Stackelberg game theory that describes the interactions between government agencies (the upper-level leader) and pharmaceutical companies (the lower-level followers) and considers the interests of patients and the sustainability of pharmaceutical R&D. In this study, the government, with patient satisfaction as the main goal, is the leader, and pharmaceutical companies, with maximum drug revenue as the goal, are the followers. The excess profit tax adopted in this study acts directly on drug sales, increases the tax burden on companies, and weakens their incentive to raise the unit price of drugs. Finally, under the background of an excess profit tax policy, medical insurance payment policies should be implemented, and key drugs related to people’s life and health should be included in the reimbursement catalog, to further reduce the burden on patients and improve patient satisfaction. The results show that under the premise of ensuring sufficient incentives for patent holders, the optimal tax policy for excess profit can effectively compensate for the shortcomings of pharmaceutical patent protection, alleviate the failure of the market regulation of drug prices, improve patient satisfaction, and increase total social welfare.

This study also has several limitations. First, to facilitate the calculation and application, the process of abstract modeling has been simplified. For example, the impact of generic pharmaceutical companies is ignored, and alternative treatment programs are not considered. Second, there are not enough data available for a validation analysis; therefore, the model cannot be further improved. Last, this paper does not consider whether companies would vote to leave the market when facing stringent tax policies.

An effective patent protection system is a necessary condition to encourage technological innovation, and a reasonable tax policy can, to a certain extent, exert a regulatory effect on drug prices. Future research could further analyze the interaction mechanisms between the patent protection system and taxes on excess profit to find a suitable balance point. Due to limited space, this study simply uses two pharmaceutical companies and does not analyze the competition between many pharmaceutical companies. In subsequent work, the model needs to be further improved to be more realistic.

## Figures and Tables

**Table 1:** Comparison of the prices of several popular drugs in the UK and US.

No.	Drug	Specifications	US	UK
1	Actimmune	12 bottles	$52,321	$6897
2	Daraprim	100 tablets	$99	$67
3	Cinryze	20 bottles	$44,140	$34,293
4	Chenodal	90 tablets	$42,570	$16,160
5	Juxtapid	30 tablets	$36,992	$14,836
6	Firazyr	2 injections	$32,468	$3597
7	Harvoni	21 tablets	$31,500	$12,561
8	Cuprimine	120 tablets	$31,426	$150.84

**Table 2:** Model parameters.

Parameters	Meaning	Parameters	Meaning
i	i=1,…,n drugs produced by pharmaceutical companies	$E_{0}$	Net income of pharmaceutical companies
$\alpha_{i}$	Premium ratio for drug prices; decision variable, $\alpha_{i}=\left(\alpha_{1},\ldots ,\alpha_{n}\right)$	$E_{1}^{i}$	Gross income for pharmaceutical companies from drug sales
$y_{i}$	Drug yield, $y_{i}=\left(y_{1},\ldots ,y_{n}\right)$	$E_{2}^{i}$	Excess profit that needs to be taxed
$\sigma_{i}$	Percentage of reimbursement for drugs; decision variable, $\sigma_{i}=\left(\sigma_{i},\ldots ,\sigma_{n}\right)$	$s_{i}$	Patient satisfaction (100 points)
t	Tax rate for excess profit by the government; decision variable	$\beta_{i}$	Weighted average coefficient, *i* = 1, 2, 3
$p_{i}$	The price of the ith drug, $p_{i}=\alpha_{i}c_{i}$	$p_{i}^{\prime }$	The actual price paid by patients for the *i*th drug
$u_{i}^{1}$	Lagrange multiplier	$u_{i}^{2}$	Lagrange multiplier
$\nu_{1}$	Lagrange multiplier	$\nu_{2}$	Lagrange multiplier

**Table 3:** The relationship between enterprise profit and financial revenue under high satisfaction.

Satisfaction	Profit	Finance	Satisfaction	Profit	Finance
99.98786891	5,049,000	688,911,300	65.05754476	1,626,500	143,276,000
87.13054187	163,700	4,886,000	64.99625562	4,033,700	490,212,800
80.19233752	345,976,300	347,984,000	64.94021474	1,740,600	156,222,900
79.22240803	4,978,100	673,876,400	64.88891543	3,947,200	474,855,500
75.22264631	292,200	15,063,300	64.84192037	1,853,500	169,353,800
72.80927835	4,906,000	658,773,500	64.79885057	3,859,500	459,523,800
71.19565217	419,500	25,511,000	64.75937641	1,965,200	182,661,500
70.04147813	4,832,700	643,609,800	64.72321032	3,770,600	444,224,900
69.79199662	14,857,900	681,953,600	64.69010074	2,075,700	196,138,800

## Data Availability

Not applicable.
